# Deaths among tuberculosis cases in Shanghai, China: who is at risk?

**DOI:** 10.1186/1471-2334-9-95

**Published:** 2009-06-17

**Authors:** Xin Shen, Kathryn DeRiemer, Zheng'an Yuan, Mei Shen, Zhen Xia, Xiaohong Gui, Lili Wang, Jian Mei

**Affiliations:** 1 Department of TB Control, Shanghai Municipal Center for Disease Control and Prevention, Shanghai, PR China; 2 School of Medicine, University of California, One Shields Avenue, Davis, California, USA

## Abstract

**Background:**

Information about the risk factors associated with death caused by tuberculosis (TB) or death with TB would allow improvements in the clinical care of TB patients and save lives. The present study sought to identify characteristics associated with increased risk of death during anti-TB treatment in Shanghai, a city in China with one of the country's highest TB mortality rates.

**Methods:**

We evaluated deaths among culture positive pulmonary TB cases that were diagnosed in Shanghai during 2000–2004 and initiated anti-TB therapy. Demographic, clinical, mycobacteriological information and treatment outcomes were routinely collected through a mandatory reporting system.

**Results:**

There were 7,999 culture positive pulmonary cases reported during the study period. The overall case fatality rate was 5.5% (440 cases), and approximately half (50.5%) of the deaths were attributed to causes other than TB. Eighty-six percent of the deaths were among TB cases age ≥ 60 years. The significant independent risk factors for mortality during anti-TB treatment were advancing age, male sex, sputum smear positivity, and the presence of a comorbidity.

**Conclusion:**

More vigorous clinical management and prevention strategies by both the TB control program and other public health programs are essential to improve TB treatment outcomes. Earlier suspicion, diagnosis and treatment of TB, especially among persons older than 60 years of age and those with a comorbid condition, could reduce deaths among TB patients.

## Background

Globally, tuberculosis (TB) caused 1.8 million deaths in 2007 [1], and TB is among the top ten causes of death worldwide, especially in Asia and Africa [1,2]. Death is the worst possible outcome for a person with TB. Information about the determinants of death among TB patients could help identify individuals who are at higher risk of death so that targeted interventions can be implemented and TB treatment outcomes can be improved. Risk factors such as older age [3-5], a history of prior TB treatment [3,6], multi-drug resistance (MDR) [3,6], HIV [4,7], comorbidity [8], and incomplete treatment [9] are known to increase the mortality associated with TB. However, the risk factors for death among TB cases in China are unknown.

China reported the second-highest number of new TB cases (1.31 million) and the second-highest number of TB deaths (201,000 TB cases) in 2007, behind only India [1]. Shanghai is a metropolitan area located on the east coast of China. Although the notification rate of pulmonary TB in Shanghai (39.4/100 000 in 2000) [10] was lower than the national rate (41.7/100 000 in 2000) [11], mortality among TB patients in Shanghai (2.22/100 000 in 2000) [10] was 56 times higher than the average national mortality rate among TB patients (0.04/100 000 in 2000) [11]. Of all of the notifiable communicable diseases in Shanghai, TB ranked first in terms of reported deaths in 2000 [10]. In the present study, we sought to identify the risk factors associated with death among pulmonary TB patients during anti-TB treatment in Shanghai.

## Methods

### Study area

Shanghai is divided into 19 administrative regions. The Shanghai Municipal Center for Disease Control and Prevention (Shanghai CDC) manages the TB Control Program for the municipality of Shanghai, and each of 19 regions in Shanghai has their own local government and health activities, including the local TB control program. There were approximately 16 million persons in Shanghai in 2000, including 13.2 million local residents and an estimated 3 million urban migrants (migrants from rural areas in other provinces in China). The estimated population density in Shanghai is over 2500 persons per square kilometer.

### TB surveillance

Beginning in the 1990s, a TB surveillance system and mandatory reporting system for all pulmonary TB patients were implemented in Shanghai. All suspected cases of pulmonary TB at each hospital or clinic in Shanghai were referred to a specialized TB hospital or TB clinic, where demographic, clinical, and mycobacteriological information, and the treatment outcome for each patient was collected and reported to the TB Registry at Shanghai CDC. While the treatment outcomes for Shanghai residents with pulmonary TB patient were routinely reported to the TB Registry, treatment outcomes for pulmonary TB patients among urban migrants were not reported until 2005. As culture remains the "gold standard" for TB diagnosis, and is essential for traditional drug susceptiblity testing, we used all culture-positive pulmonary TB patients among local residents reported in Shanghai during 2000–2004 as our study population.

### Laboratory methods

Three sputum specimens were routinely collected from each suspect TB case. Those individuals with bacteriological confirmation of *Mycobacterium tuberculosis (M. tuberculosis) *in their smear and culture initiated anti-TB treatment at a TB hospital or clinic. All of the pretreatment positive cultures of *M. tuberculosis *in each laboratory were sent to the Tuberculosis Reference Laboratory at Shanghai CDC for drug susceptibility testing and specimen identification. Drug susceptibility testing was routinely performed on isolates of *M. tuberculosis *for isoniazid, rifampin, ethambutol and streptomycin on Lowenstein-Jensen (LJ) solid medium [12].

### Treatment

In general, new pulmonary TB patients received two months of treatment with isoniazid (H), rifampin (R), pyrazinamide (Z), and ethambutol (E) (or streptomycin (S)) during the intensive phase and four months of isoniazid and rifampin during the continuation phase (2HRZE(S)/4HR). Retreatment pulmonary TB patients received treatment using 2HRZES/1HRZE/5HRE or 2HRZES/6HRE. Both the intensive phase and the continuation phase of anti-TB therapy could be extended, depending on radiological and bacteriological data that were available and the clinical judgment of the treating physician.

### Definitions

A suspected case of pulmonary TB was defined as an individual with an unexplained productive cough during the previous 2–3 weeks. A TB case was defined as an individual who was sputum smear positive or culture positive for *M. tuberculosis*. A new TB case was defined as a TB case without a previous history of TB treatment. A retreatment TB case was defined as a TB case who had previously received at least 30 days of anti-TB treatment any time during their life. MDR was defined as resistance to at least isoniazid and rifampin. Treatment success and transfer out of the district were defined according to international definitions [13]. The cause of death was determined based on the information from the death certificate. A TB death was defined as a TB case whose treatment outcome was recorded as death caused by TB, or death with TB as a contributing cause. All remaining deaths were defined as non-TB deaths.

In the present study, TB cases were followed from the time they initiated treatment until they stopped treatment for any reason, such as a death, default, transfer, move, or successful completion of therapy. However, we excluded from the analysis those TB cases whose treatment extended beyond 12 months, those who moved or transferred out of Shanghai, and those for whom no information was available about the treatment outcome. Treatment outcomes for pulmonary TB patients among urban migrants were not reported until 2005 and they were also excluded from the analysis. Comorbidity was defined as a chronic illness, such as diabetes, cardiovascular disease, cancer, or chronic obstructive pulmonary disease, or a concurrent infectious disease such as hepatitis or pneumonia. In this study, comorbidity was recorded as the presence or absence of a comorbidity, but precise information was not collected about the actual disease condition.

### Statistical analysis

We used univariate and multivariate analyses to determine the characteristics of the TB cases that were associated with an increased risk of all cause mortality during anti-TB treatment. The independent variables studied included demographic characteristics (i.e., age, sex), clinical characteristics (i.e., previous TB history, cavity on initial chest radiograph, comorbidity), and sputum smear status. All independent variables were examined as categorical variables. Odd ratios (ORs) and 95% confidence intervals (CI) were calculated to measure the association between TB patient characteristics and death, at the univariate and multivariate level. All variables with a *p *value < 0.20 in the univariate analysis were considered for the multivariate logistic regression model. Forward stepwise model construction was performed and the log likelihood ratios of successive models were compared until the final, most parsimonious model was identified. We tested for interaction between those variables with a *p *value < 0.05 in the multivariate model, and kept the interaction terms in the multivariate model if they were significant (*p *< 0.05). All analyses used Stata statistical software (version 8.0SE, Stata Corporation, College Station, Texas, USA).

### Ethical approval

The study was approved by the Committee for Medical Research Ethics at Shanghai CDC and the University of California, Davis.

## Results

There were a total of 21,292 pulmonary TB cases reported in Shanghai during 2000–2004. Of these, 15,035 (70.6%) had a sputum culture result available and 8,018 of the TB cases were sputum culture positive. TB cases who were male (72.0% versus 67.2%; p < 0.001), had cavity on initial chest radiograph (74.3% versus 70.0%; p < 0.001), and had a comorbidity (72.9% versus 70.3%, p = 0.002) were more likely to have sputum culture results available. We also tested the proportion of other subgroups (by age group, sputum smear status, new cases versus retreatment cases), but no significant differences were observed between TB cases who did and did not have a culture result.

**Figure 1:**
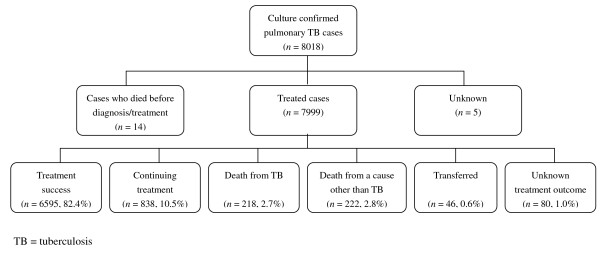
**Study population**.

Of the 8,018 culture-positive pulmonary TB cases, 7,999 cases initiated anti-TB treatment (Figure 1). Of the 7,999 cases initiated anti-TB treatment, 16.9% were followed for up to 6 months, 49.2% were followed for up to 9 months, and 33.9% were followed for up to 12 months. The median follow up period was 229 days for all cases, 219 days for new cases, and 272 days for retreatment cases (p < 0.001). At the end of one year follow-up, a total of 7,433 (92.9%) treated cases were alive (6,595 were considered a treatment success, and 838 were continuing treatment), and 440 (5.5%) cases had died prior to completion of therapy. One hundred and twenty-six cases had either transferred out of Shanghai or their treatment status was unknown, and they were excluded in the analysis. Among the TB cases who died, 49.5% (218/440) of the deaths were directly caused by TB, and 50.5% (222/440) were caused by a cause other than TB. Among new TB cases, 49.8% of all deaths were attributable to causes other than TB, while among retreatment TB cases, 46.7% of all deaths were attributable to causes other than TB (p = 0.441). In addition, 85.7% of the deaths were among TB cases age ≥ 60 years (Table 1).

**Table 1:** Characteristics of the 7,873 study participants with tuberculosis (TB), Shanghai, 2000–2004

Characteristics	Total		Survived		Died	
	No.	(%)	No.	(%)	No.	(%)

**Age (years)**						
Median (range, years)	52	(11–100)	51	(11–100)	74	(19–92)
11–14	33	(0.4)	33	(0.4)	0	(0.0)
15–29	1003	(12.7)	1001	(13.5)	2	(0.5)
30–44	1643	(20.9)	1624	(21.8)	19	(4.3)
45–59	1966	(25.0)	1924	(25.9)	42	(9.5)
60–74	2057	(26.1)	1883	(25.3)	174	(39.5)
≥ 75	1171	(14.9)	968	(13.0)	203	(46.1)
**Sex**						
Female	1736	(22.1)	1681	(22.6)	55	(12.5)
Male	6137	(77.9)	5752	(77.4)	385	(87.5)
**Case type**						
New case	6383	(81.1)	6056	(81.5)	327	(74.3)
Retreatment case	1490	(18.9)	1377	(18.5)	113	(25.7)
**Cavity on initial chest radiograph**						
Absent	5258	(66.8)	4943	(66.5)	315	(71.6)
Present	2376	(30.2)	2266	(30.5)	110	(25.0)
Uncertain	239	(3.0)	224	(3.0)	15	(3.4)
**Sputum smear**						
Negative	1461	(18.6)	1416	(19.0)	45	(10.2)
Positive	6334	(80.4)	5945	(80.0)	389	(88.4)
Unkown	78	(1.0)	72	(1.0)	6	(1.4)
**Comorbidity**						
Absent	6231	(79.1)	5938	(79.9)	293	(66.6)
Present	1608	(20.4)	1466	(19.7)	142	(32.3)
Unknown	34	(0.4)	29	(0.4)	5	(1.1)
**Drug susceptibility results**						
Non-MDR	4714	(59.9)	4487	(60.4)	227	(51.6)
MDR	256	(3.2)	241	(3.2)	15	(3.4)
Unknown	2903	(36.9)	2705	(36.4)	198	(45.0)

TB = tuberculosis; MDR = multidrug resistance

The characteristics of the study population are presented in Table 1. The median age of all TB cases in the study was 52 years. TB cases were predominantly male (77.9%). Most TB cases were new cases (81.1%). Thirty percent of all of the TB cases had a cavity on their initial chest radiograph and 80.4% were sputum smear positive. Twenty percent of all TB cases had a comorbid condition, and 3.2% of the TB cases had MDR.

### Risk factors for on-treatment mortality

Based on the univariate analysis, the characteristics of the TB patients associated with mortality during anti-TB treatment were age 30–44 years, age 45–59 years, age 60–74 years, age ≥ 75 years, male sex, being a retreatment case, the presence of a cavity on the initial chest radiograph, sputum smear positivity, and the presence of a comorbidity (Table 2). In the final multivariable logistic regression model (Table 2), TB cases with age 30–44 years (adjusted OR = 5.0, 95% CI: 1.2–21.7), age 45–59 years (adjusted OR = 8.2, 95% CI: 2.0–34.3), age 60–74 years (adjusted OR = 35.4, 95% CI: 8.7–143.6), age ≥ 75 years (adjusted OR = 82.3, 95% CI: 20.3–333.2), males (adjusted OR = 1.7, 95% CI: 1.2–2.3), those with a postive sputum smear result (adjusted OR = 1.6, 95% CI: 1.2–2.2), and those who had a comorbidity (adjusted OR = 1.4, 95% CI: 1.1–1.7) were more likely to die during anti-TB treatment. None of the interaction terms were significant in the multivariable logistic regression model, and were removed.

**Table 2:** Results of the univariate and multivariate analysis of the characteristics of tuberculosis (TB) patients associated with death during anti-TB treatment, Shanghai, 2000–2004

Characteristics	Survived		Died		OR	(95% CI)	*P *value	AOR	(95% CI)	*P *value
	No.	(%)	No.	(%)						
**Age (years)**										
11–29	1034	(13.9)	2	(0.5)	1.0			1.0		
30–44	1624	(21.8)	19	(4.3)	6.0	(1.5–53.6)	0.006	5.0	(1.2–21.7)	0.031
45–59	1924	(25.9)	42	(9.5)	11.3	(2.9–96.4)	<0.001	8.2	(2.0–34.3)	0.004
60–74	1883	(25.3)	174	(39.5)	47.8	(13.0–398.1)	<0.001	35.4	(8.7–143.6)	<0.001
≥ 75	968	(13.0)	203	(46.1)	108.4	(29.5–902.7)	<0.001	82.3	(20.3–333.2)	<0.001
**Sex**										
Female	1681	22.6	55	12.5	1.0			1.0		
Male	5752	77.4	385	87.5	2.0	(1.5–2.8)	<0.001	1.7	(1.2–2.3)	0.001
**Case type**										
New case	6056	81.5	327	74.3	1.0			-		
Retreatment case	1377	18.5	113	25.7	1.5	(1.2–1.9)	<0.001	-		
**Cavity on initial chest radiograph**										
Absent	4943	66.5	315	71.6	1.0			-		
Present	2266	30.5	110	25.0	0.8	(0.6–0.9)	0.016	-		
Uncertain	224	3.0	15	3.4	-			-		
**Sputum Smear**										
Negative	1461	18.6	1416	19.0	1.0			1.0		
Positive	6334	80.4	5945	80.0	2.1	(1.5–2.9)	<0.001	1.6	(1.2–2.2)	0.004
Unknown	78	1.0	72	1.0	-			-		
**Comorbidity**										
Absent	5938	79.9	293	66.6	1.0			1.0		
Present	1466	19.7	142	32.3	2.0	(1.6–2.4)	<0.001	1.4	(1.1–1.7)	0.004
Unknown	29	0.4	5	1.1	-			-		
**Drug susceptibility results**										
Non-MDR	4487	60.4	227	51.6	1.0			-		
MDR	241	3.2	15	3.4	1.2	(0.7–2.1)	0.450	-		
Unknown	2705	36.4	198	45.0	1.4	(1.2–1.8)	<0.001	-		

TB = tuberculosis; OR = odds ratio; AOR = adjusted odds ratio; CI = confidence interval; MDR = multidrug resistance

A high proportion of TB cases (36.9%) had missing data on MDR status. We developed a multivariable logistic regression model that was restricted to TB cases whose drug susceptibility test results were available. Characteristics of the TB patients that were significantly associated with on-treatment mortality were same as the model that included all culture-positive TB patients. We concluded that MDR was not significantly associated with on-treatment mortality in our study population (OR = 1.3, 95% CI: 0.7–2.3).

## Discussion

The case fatality rate among TB cases in Shanghai during 2000 to 2004 was 5.5%. The significant independent risk factors for on-treatment mortality among TB cases were advancing age, male sex, sputum smear positivity, and the presence of a comorbidity. These results suggest that more vigorous efforts should be made by both TB control programs and other public health programs to improve TB treatment outcomes and to reduce the mortality among TB patients.

Our analysis showed that approximately half (49.5%) of the deaths were cause by TB or TB was a contributing case, and the remainder 50.5%) were due to one or more causes other than TB. In previous studies, the proportion of deaths due to a cause other than TB was 98.6% in the United States of American and Canada [4], 86% in the Netherlands [7], 43% in England and Wales [14], 27.3% in Mexico [15], and 24.6% in Russia [3]. Interestingly, the proportions of deaths due to causes other than TB in developed countries were higher in industrialized countries than in developing countries, but there were also fewer studies conducted in developing countries. Perhaps in settings with a relatively low incidence of TB, deaths among TB patients were more likely to be attributed to an underlying medical conditions (e.g., older age, illness with a comorbidity, HIV, alcohol use), but not TB. In contrast, in high-incidence settings, TB was more common among younger individuals and ongoing transmission of *M. tuberculosis *was likely to occur. The high proportion (50.5%) of deaths among TB cases in Shanghai that were due to causes other than TB suggests that TB cases may require more intensive medical evaluation and care than is usually provided by traditional TB control programs. The finding also suggests that TB control programs should interact with other public health programs, since such deaths may not be preventable by improvements in the TB services alone.

In our study, advancing age was strongly associated with on-treatment mortality and is a likely confounder of the association of male sex, sputum smear status and comorbidities with on-treatment mortality. Given the increasing numbers of elderly persons in Shanghai (11.5% of the entire population was aged ≥ 65 years in 2000), TB mortality among the elderly has been an enormous concern. The aging population in Shanghai could be one of the reasons why the case fatality rate of TB cases was high. Generally, older patients experience unfavorable living conditions, malnutrition, comorbidities and less access to health care, any of which could increase the risk of death. It is possible that older TB cases had a high mortality rate because they were more likely to present with nonspecific symptoms, which may contribute to delays in diagnosis and treatment of TB and, ultimately, a higher risk of death [16,17]. More vigorous clinical management and prevention strategies including earlier suspicion, diagnosis and treatment of TB may reduce deaths among older patients. In addition, elderly people may present with more extensive TB disease, based on the initial chest radiograph [16,18].

Male sex was significantly associated with mortality in our study as well as in some previously studies [6,7,19]. A recent analysis of European surveillance data showed that male TB patients had approximately 50% higher risk of death [6]. However, other studies failed to detect a significant association between male gender and death among TB patients [4,5,20]. The higher risk in male patients was explained by some authors as the consequence of low compliance with anti-TB therapy, leading to repeated, short interruptions of treatment, or a greater occurrence of defaulting from treatment. Better case-holding strategies, such as directly observed therapy (DOT), may improve treatment outcomes [6,21].

The association between sputum smear positivity and on-treatment mortality was notable in our study. To our knowledge, this finding has not been previously reported. We speculate that patients who were sputum smear positive had a higher bacillary load, suffered from more severe TB, and had difficulty with their treatment. Further evidence is needed to support this speculation.

Comorbidity was previously reported as an important predictor of on-treatment mortality among TB patients [5,8,20]. The most common diseases that were listed with TB as the cause of death included HIV/AIDS, renal diseases, liver disease, cardiovascular disease, cancer, chronic obstructive pulmonary disease (COPD), and diabetes [3,4,7,8,20,22]. However, the effect of underlying diseases other than HIV/AIDS on the risk of death due to TB has not been studied extensively. Some diseases, such as renal disease and liver disease, may change the presentation of TB, making it more difficult to diagnose and treat and they may be associated with increased risk of toxicity caused by anti-TB drugs [23]. In our study, comorbidities were significantly associated with death among TB cases. Although we did not know the actual disease that caused death, TB screening among patients with other conditions which increase the risk of deaths might be helpful to detect TB early and to improve TB treatment outcomes.

Our study had several limitations. First, we determined the cause of death using death certificate data. In other settings, death certificates were at least partially unreliable [24]. However, in Shanghai, a death registration system was established during the 1990s based on the World Health Organization's International Classification of Disease (ICD) to classify the causes of death. Although the TB registry was separate from the death registration system in Shanghai, the death reports of these two systems are conducted by same local institutes. While it is possible that some misclassification of TB deaths or non-TB deaths occurred, the misclassification bias should have been minimized by the death registration system. Second, the proportion (36.9%) of missing data on MDR status, which was a strong predictor of death in other studies [3,6], was high in our study, and the prevalence of MDR might have been underestimated. However, in a multivariate logistic regression model based on those pulmonary TB patients who had a drug susceptibility test result available, MDR was not significantly associated with death during anti-TB treatment. Third, we excluded the TB cases among urban migrants from our analysis, as their treatment outcomes were not reported during the study period. However, migrant TB patients in Shanghai were commonly young, and deaths among them occurred infrequently (in 2006, only 8 cases were known to have died from TB). We assumed that most of the burden of TB deaths in Shanghai were among the local residents. Finally, HIV status, which was strongly associated with death among TB patients in other studies, was not available in our study. However, another ongoing study in Shanghai determined that the prevalence of HIV among TB patients is very low (<0.1%). We assumed that HIV infection was not significantly associated with death among TB patients in Shanghai.

## Conclusion

A substantial number of deaths occurred among pulmonary TB patients during anti-TB treatment in Shanghai, but a high proportion of deaths among pulmonary TB cases in Shanghai were attributable to causes other than TB. Both TB control programs and other public health programs should be strengthened and should interact with each other to improve the TB treatment outcomes and to prevent unnecessary deaths. Patients with advancing age, male sex, sputum smear positivity, and a comorbidity have the highest risk of death during anti-TB treatment. Our findings highlight the importance of improved clinical management and prevention strategies that target TB patients at increased risk of death. Further studies are needed to identify and evaluate interventions that reduce mortality among TB patients.

## Competing interests

The authors declare that they have no competing interests.

## Authors' contributions

XS, ZY, and JM participated in the study design and management and analysis of data and wrote the manuscript. KdR participated in the data analysis and writing the manuscript. MS, and ZX participated in the study design and performed data analysis. XG and LW performed the laboratory work. All authors read and approved the final manuscript.

## Pre-publication history

The pre-publication history for this paper can be accessed here:

http://www.biomedcentral.com/1471-2334/9/95/prepub
